# Incidental irradiation of internal mammary lymph nodes in breast cancer: conventional two-dimensional radiotherapy versus conformal three-dimensional radiotherapy*

**DOI:** 10.1590/0100-3984.2015.0003

**Published:** 2016

**Authors:** Elton Trigo Teixeira Leite, Rafael Tsuneki Ugino, Marco Antônio Santana, Denis Vasconcelos Ferreira, Maurício Russo Lopes, Edilson Lopes Pelosi, João Luis Fernandes da Silva, Heloisa de Andrade Carvalho

**Affiliations:** 1 MD, Radiation Oncologist, Radiation Oncology Department - Hospital Sírio-Libanês; Radiation Oncology Department - Instituto do Câncer do Estado de São Paulo Octavio Frias de Oliveira (Icesp), São Paulo, SP, Brazil.; 2 MD, Radiation Oncologist, Radiation Oncology Department - Hospital Sírio-Libanês, São Paulo, SP, Brazil.; 3 MD, Radiation Oncologist, Department of Radiology and Oncology - Faculdade de Medicina da Universidade de São Paulo (FMUSP), São Paulo, SP, Brazil.; 4 MD, PhD, Department of Radiology and Oncology - Faculdade de Medicina da Universidade de São Paulo (FMUSP), São Paulo, SP, Brazil.

**Keywords:** Lymph nodes/radiation effects, Lymphatic irradiation, Breast neoplasms, Radiotherapy

## Abstract

**Objective:**

To evaluate incidental irradiation of the internal mammary lymph nodes (IMLNs) through opposed tangential fields with conventional two-dimensional (2D) or three-dimensional (3D) radiotherapy techniques and to compare the results between the two techniques.

**Materials and Methods:**

This was a retrospective study of 80 breast cancer patients in whom radiotherapy of the IMLNs was not indicated: 40 underwent 2D radiotherapy with computed tomography for dosimetric control, and 40 underwent 3D radiotherapy. The total prescribed dose was 50.0 Gy or 50.4 Gy (2.0 or 1.8 Gy/day, respectively). We reviewed all plans and defined the IMLNs following the Radiation Therapy Oncology Group recommendations. For the IMLNs, we analyzed the proportion of the volume that received 45 Gy, the proportion of the volume that received 25 Gy, the dose to 95% of the volume, the dose to 50% of the volume, the mean dose, the minimum dose (Dmin), and the maximum dose (Dmax).

**Results:**

Left-sided treatments predominated in the 3D cohort. There were no differences between the 2D and 3D cohorts regarding tumor stage, type of surgery (mastectomy, breast-conserving surgery, or mastectomy with immediate reconstruction), or mean delineated IMLN volume (6.8 vs. 5.9 mL; *p* = 0.411). Except for the Dmin, all dosimetric parameters presented higher mean values in the 3D cohort (*p* < 0.05). The median Dmax in the 3D cohort was 50.34 Gy. However, the mean dose to the IMLNs was 7.93 Gy in the 2D cohort, compared with 20.64 Gy in the 3D cohort.

**Conclusion:**

Neither technique delivered enough doses to the IMLNs to achieve subclinical disease control. However, all of the dosimetric parameters were significantly higher for the 3D technique.

## Abstract (pt)

**Objetivo:**

Avaliar a irradiação incidental dos linfonodos da cadeia mamária interna (LCMIs) com campos tangenciais opostos por meio de radioterapia bidimensional (2D) convencional ou tridimensional (3D) e comparar as duas técnicas quanto aos resultados obtidos.

**Materiais e Métodos:**

Trata-se de um estudo retrospectivo com 80 pacientes com câncer de mama sem indicação de radioterapia dos LCMIs: 40 foram submetidos a radioterapia 2D com tomografia computadorizada para controle dosimétrico e 40 foram submetidos a radioterapia 3D. A dose total prescrita foi 50,0 Gy ou 50,4 Gy (2,0 ou 1,8 Gy/dia, respectivamente). Os planos de tratamento foram analisados e os LCMIs foram definidos conforme as recomendações do Radiation Therapy Oncology Group. No tocante aos LCMIs, foram analisadas a proporção do volume que recebeu 45 Gy, a proporção do volume que recebeu 25 Gy, a dose para 95% do volume, a dose para 50% do volume, a dose média, a dose mínima (Dmín) e a dose máxima (Dmáx).

**Resultados:**

Tratamentos do lado esquerdo predominaram na coorte 3D. Não houve diferenças entre as coortes 2D e 3D quanto ao estágio do tumor, ao tipo de cirurgia (mastectomia, cirurgia conservadora ou mastectomia com reconstrução imediata) ou à média do volume delineado dos LCMIs (6,8 vs. 5,9 mL; *p* = 0,411). À exceção da Dmín, todos os parâmetros dosimétricos apresentaram médias maiores na coorte 3D (*p* < 0,05). A mediana da Dmáx na coorte 3D foi 50,34 Gy. No entanto, a dose média nos LCMIs foi 7,93 Gy na coorte 2D e 20,64 Gy na coorte 3D.

**Conclusão:**

Nenhuma das duas técnicas emitiu doses suficientes aos LCMIs para que se alcançasse o controle subclínico da doença. No entanto, todos os parâmetros dosimétricos foram significativamente maiores com a técnica 3D.

## INTRODUCTION

For more than two decades, the standard of care for patients with early stage breast cancer has been breast-conserving therapy. Radiation therapy (RT), with or without a boost to the surgical bed, plays an important role in the adjuvant scenario by improving local control^(1-8)^. The overall survival benefit of adjuvant RT for breast cancer patients was established in studies conducted more than 15 years ago^(8,9)^. In those studies, however, radiation was delivered to the surgical bed and to all corresponding lymphatic drainage regions, including the axilla, supraclavicular fossa, and internal mammary lymph nodes (IMLNs). In patients with early stage tumors, conservative treatment is indicated, with no recommendation for irradiation of the lymph nodes, and therefore little is known about the effect that regional RT has on local control and overall survival. However, some studies have identified situations in which the lymph nodes, including the IMLNs, should be irradiated following conservative surgery^(10-13)^.

In general, IMLN involvement is related to the axillary lymph node status and the location of the tumor in the breast. The IMLNs are involved in 18-52% of patients with positive axillary lymph nodes, regardless of the primary tumor location, compared with 0-16% for those with no axillary disease; among patients with tumors located in the inner quadrants, IMLN involvement occurs in 25-65% and 0-20% of those with positive and negative axillary lymph nodes, respectively^(14-16)^. Nevertheless, the rate of clinically detected recurrences in the IMLNs after primary breast treatment, mainly in the early stages, is < 10%^(17-19)^. Some authors suggest that this can be explained by incidental irradiation of the IMLN through opposed tangential fields^(13,20)^.

An abstract presented at the American Society of Clinical Oncology annual meeting in 2011^(21)^ demonstrated increased disease-free survival in response to conservative surgery and locoregional RT including all the lymphatic chains in patients with breast cancer. Despite that improvement, the wide lymphatic irradiation did not allow treatment tailoring, leading the authors to improve their selection of the target volume regarding the lymph nodes: the supraclavicular fossa only; the IMLNs only; or both. In the few randomized studies published on this subject, no difference was observed between the studied groups in terms of local control or overall survival^(11,22,23)^. However, higher pulmonary toxicity is expected when the IMLNs are included in the irradiation fields. Therefore, IMLN irradiation is still controversial.

The RT target volumes are well defined for conventional two-dimensional (2D) and conformal three-dimensional (3D) RT techniques^(24,25)^. When the 3D technique is used, there can be incidental irradiation of certain areas, including the IMLNs. Incidental irradiation of the axillary lymph nodes through opposed tangential fields reportedly occurs in up to 22.3% of cases^(26)^, although there have been no reports of such irradiation of the IMLNs.

As previously mentioned, the inclusion of the lymphatic drainage regions in the RT of breast cancer has the potential to improve survival. Therefore, the purpose of this study was to evaluate the incidental irradiation of the IMLNs in patients submitted to conventional 2D or conformal 3D RT, with no formal recommendation for irradiation of the IMLNs, as well as to compare the two treatment techniques in terms of the results obtained.

## MATERIALS AND METHODS

This was a retrospective study of 80 breast cancer patients who underwent breast surgery and 2D or 3D RT between January and March 2012. Of those 80 patients, 40 were treated at a public facility and 40 were treated at a private facility. Consecutive patients were selected, and we included cases with no formal recommendation for irradiation of the IMLNs. The site of treatment, tumor stage, and type of surgery (mastectomy, breast-conserving surgery, or mastectomy with immediate reconstruction) were recorded for posterior comparisons.

For the patients treated at the public facility, the 2D technique with computed tomography for RT planning was employed, whereas the 3D RT technique was employed for those treated at the private facility. The total prescribed dose was 50.0 Gy or 50.4 Gy, in conventional fractions of 2.0 or 1.8 Gy/day, respectively. In the 2D simulation, patients were immobilized with a breast board and the field borders (the medial border, at the midline; the lateral border, at the midaxillary line; the upper border, at the second intercostal space or including the whole breast with a 1-cm margin; and the lower border, 1-2 cm below the inferior breast fold) were marked on the skin. The beam angles were defined according to those marks, and the lung volumes were included in the tangential fields- < 3 cm (preferentially < 2 cm) in the isocenter plane. The planning system was then optimized in order to achieve better dose distribution. In the 3D simulation, patients were immobilized with a vacuum cushion and the planning of the tangential fields was based on the target volume delineation for the dose-volume distribution analysis. In both techniques, the goal was better dose homogeneity-dose in the target volume ranging from -5% to +7%, in accordance with the ICRU 50 recommendations^(27)^. Wedges and field-in-field strategies were used.

**Figure 1:**
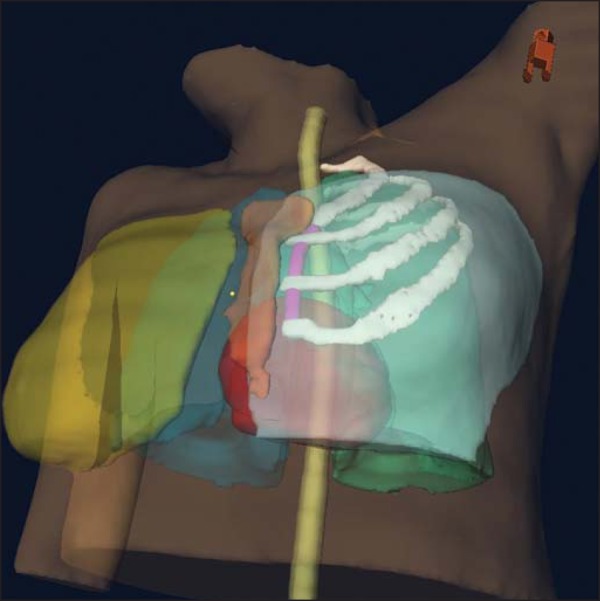
Internal mammary lymph node volume (pink): first, second, and third intercostal spaces.

**Figure 2:**
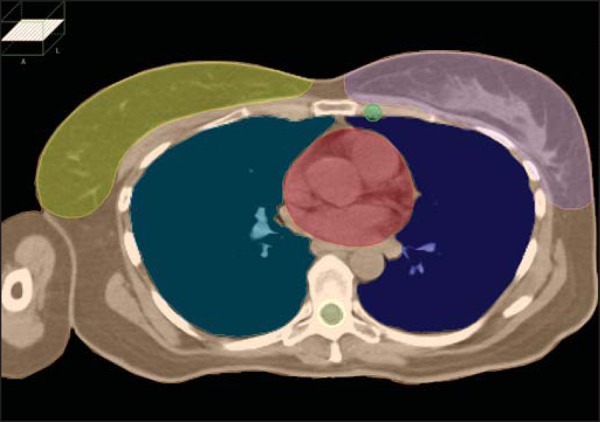
Volumes (lungs, heart, spinal cord, contralateral breast, clinical target, and internal mammary lymph nodes) contoured in accordance with RTOG Breast Cancer Atlas for Radiation Therapy Planning: Consensus Definitions.

We reviewed all plans and defined the IMLNs following the Radiation Therapy Oncology Group (RTOG) recommendations^(25)^: inclusion of the IMLNs from the superior aspect of the medial first rib to the cranial aspect of the fourth rib (Figure 1), ipsilateral to the treatment site, with a radial margin of 0.5 cm (Figure 2). After delineation, the IMLN volumes irradiated through the tangential fields were evaluated for both techniques (2D and 3D), as follows: proportion of the volume receiving at least 45 Gy (V45, the minimal dose required for subclinical disease control); dose to 95% of the volume (D95); minimum dose (Dmin); maximum dose (Dmax); mean dose (Dmean); the volume receiving at least 25 Gy (V25), corresponding to the field borders; and the dose to 50% of the IMLN volume (D50). The IMLN delineation results were also compared between both techniques.

Few patients received irradiation of the supraclavicular fossa or a boost to the surgical bed. However, those fields were not considered in the final calculation and dosimetric evaluation.

## RESULTS

**Figure 3:**
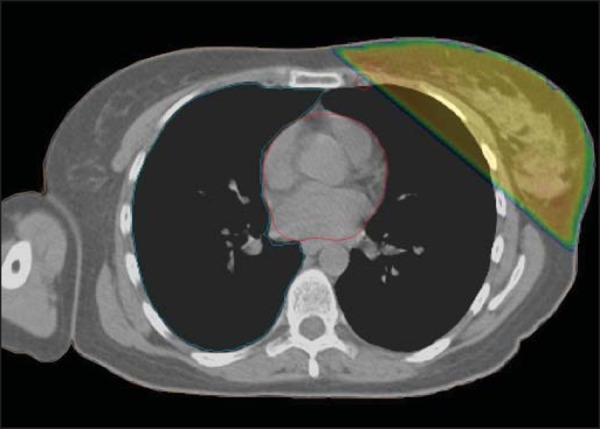
Internal mammary lymph nodes (red) and isodose curve (45 Gy; green).

Left-sided treatments predominated in the 3D cohort. There were no statistically significant differences between the 2D and 3D techniques regarding tumor stage, type of surgery (Table 1), or mean IMLN contouring volume (6.8 mL vs. 5.9 mL; *p* = 0.411). Except for the Dmin, all of the dosimetric parameters analyzed (V45, D95, Dmax, Dmean, V25, and D50) presented higher mean values in the 3D planning (*p* < 0.05). In the 3D planning, the median Dmax was 50.34 Gy, although the IMLNs received a mean of 20.64 Gy, the V45 for the IMLNs being only 15.8% (Figure 3). Contouring and dosimetric results are depicted in Table 2.

**Table 1:** Characteristics of the two cohorts studied.

Characteristic	2D	3D	*P*
Treatment side			
Right	22 (55.0%)	13 (32.5%)	0.033
Left	18 (45.0%)	27 (67.5%)	
Tumor stage			
0	4 (10.0%)	8 (20.0%)	
IA	6 (15.0%)	26 (65.0%)	
IIA	11 (27.5%)	2 (5.0%)	
IIB	9 (22.5%)	0	0.093
IIIA	7 (17.5%)	0	
IIIB	1 (2.5%)	2 (5.0%)	
IIIC	2 (5.0%)	0	
Type of surgery			
Breast-conserving	23 (57.5%)	35 (87.5%)	
Mastectomy	11 (27.5%)	0	0.187
Breast reconstruction	6 (15.0%)	5 (12.5%)	

**Table 2:** Comparison of the delineated volumes of the internal mammary lymph nodes and the dosimetric parameters between the 2D RT technique (performed at a public facility) and the 3D RT technique (performed at a private facility).

	2D			3D		
Parameter	Median (min–max)	Mean (SD)		Median (min–max)	Mean (SD)	*P*
IMLN volume (mL)	6.8 (5–8.1)	6.8 (0.82)		5.7 (4.4–8.0)	5.9 (0.97)	0.411
V45 (%)	0 (0–27)	2.2 (5.6)		7.5 (0–96)	15.8 (23.5)	0.020
D95 (cGy)	196.6 (44–742)	228.1 (136.6)		533.5 (242–4609)	753 (766)	< 0.0001
Dmin (cGy)	181.4 (37–369)	1790 (80.2)		470 (220–4414)	639.5 (708)	< 0.0001
Dmax (cGy)	354.3 (108–5358)	2827.7 (1981.5)		5034 (756–5541)	4198.3 (1499)	< 0.0001
Dmean (cGy)	508.25 (70–3245)	793.5 (772.6)		1811.5 (438–5295)	2064.2 (1331)	< 0.0001
V25 (%)	0.35 (0–53.7)	7.8 (13.8)		27.6 (0–99.6)	31.9 (29.8)	< 0.0001
D50 (cGy)	350.8 (73–3430)	619.7 (738.9)		1193 (50-5332)	1746.7 (1459.5)	< 0.0001

IMLN, Internal mammary lymph nodes; V45, proportion of the volume receiving at least 45 Gy; D95, dose to 95% of the volume; Dmin, minimum dose; Dmax, maximum dose; Dmean, mean dose; V25, proportion of the volume receiving at least 25Gy; D50, dose to 50% of the volume; SD, standard deviation.

## DISCUSSION

Management of the IMLNs in breast cancer is still controversial. Clinical trials analyzing the surgical resection of IMLNs have shown no significant benefit in overall survival^(18,28-30)^. In studies showing that postmastectomy RT provides a benefit, all lymph node chains, including the IMLNs, were irradiated, although no benefit was found to be specifically associated with IMLN irradiation^(8,9,12)^. In a phase II study involving 100 women at high-risk (with stage II-III breast cancer) submitted to doxorubicin-based chemotherapy and locoregional RT^(31)^, there were 33 who, for technical reasons, did not receive IMLN irradiation. Comparing the patients who did and did not receive IMLN irradiation, the authors found that, over the 77 months of follow-up, the disease-free survival rate was higher in the former group (73% vs. 52%; *p* < 0.05), as was the overall survival benefit (78% vs. 64%; *p* = 0.008). A study conducted in France analyzed the role of postmastectomy IMLN irradiation in 1407 women with breast cancer in an initial stage (I-II) and showed that most (86%) of those women received systemic treatment^(32)^. The authors found no statistically significant difference between the women who received IMLN irradiation and those who did not in terms of the 10-year overall survival rate (59.3% vs. 62.6%). Cardiac toxicity was reported in 7 of the patients who received IMLN irradiation and in 5 of those who did not^(32)^. In a cohort of 2413 women with T3-4 N0 breast cancer or positive lymph nodes, evaluated between 2001 and 2006 in British Columbia, Canada^(33)^, IMLN irradiation was not found to improve locoregional control or overall survival. However, when the authors of that study evaluated only the women with positive lymph nodes, they found that overall survival was better in those who received with IMLN RT than in those who did not (91% vs. 88%; *p* = 0.01).

The role of RT in local control and survival remains controversial and has been studied by many authors. In general, there is no difference between groups receiving and not receiving IMLN irradiation concerning local control and overall survival. However, pulmonary toxicity seems to be more common in the former. The negative survival benefit data observed for IMLN RT might be due to a low risk profile in the selected population or to a tendency in the study design to detect significant (> 10%) differences in survival^(26)^. Therefore, the hypothesis that there are minor benefits in overall survival or local control, mainly in women at high risk for IMLN involvement (i.e., those with inner quadrant tumors and axillary disease), cannot be excluded. The results from the largest phase III trial of irradiation of the supraclavicular fossa and the IMLNs^(34)^, which featured a 10-year follow-up period, were presented at the European Cancer Congress 2013. The authors demonstrated a tendency of such irradiation (in comparison with whole-breast or thoracic-wall irradiation alone) to increase overall survival (82.3% vs. 80.7%; *p* = 0.056), as well as a significant benefit in dis-ease-free survival (72.1% vs. 69.1%; *p* = 0.044) and in metastasis-free survival (78% vs. 75%; *p* = 0.02), without treatment related morbidity, thus supporting the assumption that nodal irradiation provides benefits^(11,31,35)^.

The incidence of metastasis in the IMLNs varies according to the size and location of the tumor within the breast, as well as the axillary lymph node status. Therefore, for tumors with a diameter of < 0.5 cm, the incidence of such metastasis is 3-7%, whereas it is 40-60% for tumors ranging from 3.1 cm to 5 cm in diameter^(36)^. In patients with inner quadrant tumors and axillary lymph node involvement, the reported rate of IMLN involvement is 45% when the tumor is in the upper inner quadrant and 72% when it is in the lower inner quadrant^(36)^. Despite these high rates of IMLN involvement, the reported recurrence rates in the IMLN after the treatment of primary breast cancer are < 1%^(19,37,38)^. One may argue that this could be due to the incidental irradiation of the IMLNs through the classic opposed tangential fields, which could deliver enough doses to achieve subclinical disease control.

Using clinical reference points, Proulx et al.^(20)^ planned and executed treatment with standard tangential fields in 50 women who had recently undergone either lumpectomy or mastectomy. Post-planning computed tomography scans were obtained, and the tangential radiation fields were visualized through the use of radiopaque markers affixed to the skin. The results were analyzed statistically for the frequency of inclusion of the IMLNs in the tangential radiation treatment portals as determined on the computed tomography scans. Among the 50 patients, the IMLNs were found to be completely within the tangential fields in only 14%, partially within the tangential fields in 40%, and completely outside the tangential fields in the remaining 46%. However, those authors analyzed only the internal mammary vessels, did not define the clinical or planning target volume, and did not determine the dosimetry^(20)^. In a similarly designed study, Hare et al.^(12)^ showed complete or partial coverage of the IMLNs in 70% of the cases, without dosimetry. The authors suggested that this incidental irradiation partially explains the low failure rates in the IMLNs. Without a dose/volume measurement, it is difficult to ascertain the true clinical impact of incidental irradiation.

In the present study, we analyzed the true incidental dose in an RTOG-based IMLN volume in patients for whom IMLN irradiation was not specifically indicated. We quantified that irradiation with 2D and 3D techniques. The results were analyzed individually and compared between the two techniques. The mean D95 for the IMLN volume was 228.1 cGy and 753.0 cGy for the 2D and 3D techniques, respectively. The mean V45 for the IMLN volume was 2.2% and 15.8% for the 2D and 3D techniques, respectively. Therefore, the IMLN volumes did not receive a significant dose of incidental irradiation.

All dosimetric parameters are expected to be higher when the 3D technique is used than when the 2D technique is used. Although one may argue that this is related to the contouring, we found no difference between the two facilities under study in terms of contouring. In addition, most of the patients submitted to the 3D technique had early-stage breast cancer and had undergone breast-conserving surgery, unlike those submitted to the 2D technique (Table 1; *p* > 0.05). Patients treated at the public facility (i.e., those submitted to the 2D technique) presented disease that was more advanced. Consequently, mastectomy, which necessitates the inclusion of the chest wall in the radiation field, was performed in a larger number of cases at that facility. Even the fact that left-sided treatments could be more "economical", sparing the heart and reducing the IMLN coverage, might not explain our findings, because most left-sided treatments were performed in the 3D cohort. This supports the hypothesis that the higher doses observed with the 3D technique are a consequence of better planning of the target volume coverage due to better visualization of the target and of the organs at risk, as well as of the fact that the use of the 3D technique makes it possible to analyze the dose-volume histograms. In addition, neither the 3D nor the 2D technique allowed the minimal dose for subclinical disease control in the IMLNs to be attained.

## CONCLUSION

Incidental irradiation of the IMLNs through opposed tangential fields does not provide adequate doses for subclinical disease control with the 2D or 3D RT techniques.

## References

[1] Fisher B, Anderson S, Bryant J (2002). Twenty-year follow-up of a randomized trial comparing total
mastectomy, lumpectomy, and lumpectomy plus irradiation for the treatment of
invasive breast cancer. N Engl J Med.

[2] van Dongen JA, Voogd AC, Fentiman IS (2000). Long-term results of a randomized trial comparing
breast-conserving therapy with mastectomy: European Organization for
Research and Treatment of Cancer 10801 trial. J Natl Cancer Inst.

[3] Veronesi U, Cascinelli N, Mariani L (2002). Twenty-year follow-up of a randomized study comparing
breast-conserving surgery with radical mastectomy for early breast
cancer. N Engl J Med.

[4] Veronesi U, Marubini E, Mariani L (2001). Radiotherapy after breast-conserving surgery in small breast
carcinoma: long-term results of a randomized trial. Ann Oncol.

[5] Liljegren G, Holmberg L, Bergh J (1999). 10-Year results after sector resection with or without
postoperative radiotherapy for stage I breast cancer: a randomized
trial. J Clin Oncol.

[6] Forrest AP, Stewart HJ, Everington D (1996). Randomized controlled trial of conservation therapy for breast
cancer: 6-year analysis of the Scottish trial. Scottish Cancer Trials Breast
Group. Lancet.

[7] Holli K, Hietanen P, Saaristo R (2009). Radiotherapy after segmental resection of breast cancer with
favorable prognostic features: 12year follow-up results of a randomized
trial. J Clin Oncol.

[8] Overgaard M, Jensen MB, Overgaard J (1999). Postoperative radiotherapy in high-risk postmenopausal
breast-cancer patients given adjuvant tamoxifen: Danish Breast Cancer
Cooperative Group DBCG 82c randomised trial. Lancet.

[9] Overgaard M, Hansen PS, Overgaard J (1997). Postoperative radiotherapy in high-risk premenopausal women with
breast cancer who receive adjuvant chemotherapy. Danish Breast Cancer
Cooperative Group 82b Trial. N Engl J Med.

[10] Matzinger O, Heimsoth I, Poortmans P (2010). Toxicity at three years with and without irradiation of the
internal mammary and medial supraclavicular lymph node chain in stage I to
III breast cancer (EORTC trial 22922/10925). Acta Oncol.

[11] Chetty U, Jack W, Prescott RJ (2000). Management of the axilla in operable breast cancer treated by
breast conservation: a randomized clinical trial. Edinburgh Breast
Unit. Br J Surg.

[12] Hare GB, Proulx GM, Lamonica DM (2004). Internal mammary lymph node (IMN) coverage by standard radiation
tangent fields in patients showing IMN drainage on lymphoscintigraphy:
therapeutic implications. Am J Clin Oncol.

[13] Ragaz J, Olivotto IA, Spinelli JJ (2005). Locoregional radiation therapy in patients with high-risk breast
cancer receiving adjuvant chemotherapy: 20-year results of the British
Columbia randomized trial. J Natl Cancer Inst.

[14] Morimoto T, Monden Y, Takashima S (1994). Five-year results of a randomized clinical trial comparing
modified radical mastectomy and extended radical mastectomy for stage II
breast cancer. Surg Today.

[15] Chen RC, Lin NU, Golshan M (2008). Internal mammary nodes in breast cancer: diagnosis and
implications for patient management - a systematic review. J Clin Oncol.

[16] Strom EA, McNeese MD (1999). Postmastectomy irradiation: rationale for treatment field
selection. Semin Radiat Oncol.

[17] Veronesi U, Valagussa P (1981). Inefficacy of internal mammary nodes dissection in breast cancer
surgery. Cancer.

[18] Buzdar AU, McNeese MD, Hortobagyi GN (1990). Is chemotherapy effective in reducing the local failure rate in
patients with operable breast cancer?. Cancer.

[19] Zhang YJ, Oh JL, Whitman GJ (2010). Clinically apparent internal mammary nodal metastasis in patients
with advanced breast cancer: incidence and local control. Int J Radiat Oncol Biol Phys.

[20] Proulx GM, Lee RJ, Stomper PC (2001). Internal mammary lymph node inclusion in standard tangent breast
fields: effects of body habitus. Breast J.

[21] Whelan TJ, Olivotto I, Ackerman I (2011). NCIC-CTG MA.20: An intergroup trial of regional nodal irradiation
in early breast cancer. J Clin Oncol.

[22] Kaija H, Maunu P (1995). Tangential breast irradiation with or without internal mammary
chain irradiation: results of a randomized trial. Radiother Oncol.

[23] Hennequin C, Bossard N, Servagi-Vernat S (2013). Ten-year survival results of a randomized trial of irradiation of
internal mammary nodes after mastectomy. Int J Radiat Oncol Biol Phys.

[24] Recht A, Gunderson LL, Tepper JE (2012). Breast cancer: stages I and II. Clinical radiation oncology.

[25] Radiation Therapy Oncology Group Breast cancer atlas for radiation therapy planning: consensus
definitions.

[26] Bonavitacola P, Sioshansi S, Rava PS (2012). Incidental axillary coverage comparasion between 3DCRT and hybrid
IMRT for whole breast irradiation. Int J Radiat Oncol Biol Phys.

[27] Lee JW, Hong S, Choi KS (2008). Performance evaluation of fieldin-field technique for tangential
breast irradiation. Jpn J Clin Oncol.

[28] Meier P, Ferguson DJ, Karrison T (1989). A controlled trial of extended radical versus radical mastectomy.
Ten-year results. Cancer.

[29] Lacour J, Lê M, Caceres E (1983). Radical mastectomy versus radical mastectomy plus internal
mammary dissection. Ten year results of an international cooperative trial
in breast cancer. Cancer.

[30] Lacour J, Lê MG, Hill C (1987). Is it useful to remove internal mammary nodes in operable breast
cancer?. Eur J Surg Oncol.

[31] Stemmer SM, Rizel S, Hardan I (2003). The role of irradiation of the internal mammary lymph nodes in
high-risk stage II to IIIA breast cancer patients after high-dose
chemotherapy: a prospective sequential nonrandomized study. J Clin Oncol.

[32] Romestaing P, Belot A, Hennequin C (2009). Ten-year results of a randomized trial of internal mammary chain
irradiation after mastectomy. Int J Radiat Oncol Biol. Phys.

[33] Olson RA, Woods R, Speers C (2012). Does the intent to irradiate the internal mammary nodes impact
survival in women with breast cancer? A population-based analysis in British
Columbia. Int J Radiat Oncol Biol Phys.

[34] Poortmans, Collette S, Kirkove C (2015). Internal mammary and medial supraclavicular irradiation in breast
cancer. N Engl J Med.

[35] Poortmans PM, Venselaar JL, Struikmans H (2001). The potential impact of treatment variations on the results of
radiotherapy of the internal mammary lymph node chain: a quality-assurance
report on the dummy run of EORTC Phase III randomized trial 22922/10925 in
Stage I-III breast cancer(1). Int J Radiat Oncol Biol Phys.

[36] Handley RS (1975). Carcinoma of the breast. Ann R Coll Surg Engl.

[37] Galper S, Recht A, Silver B (1999). Factors associated with regional nodal failure in patients with
early stage breast cancer with 0-3 positive axillary nodes following
tangential irradiation alone. Int J Radiat Oncol Biol Phys.

[38] Stefanik D, Goldberg R, Byrne P (1985). Local-regional failure in patients treated with adjuvant
chemotherapy for breast cancer. J Clin Oncol.

